# Effect of glucocorticoids combined with disease modifying anti-rheumatic drugs on the improvement of symptoms in patients with rheumatoid arthritis

**DOI:** 10.12669/pjms.38.4.5458

**Published:** 2022

**Authors:** Lulu Si, Yunyan Jin, Dongni Zhao, Lixia Yu, Huankun Cao

**Affiliations:** 1 Lulu Si, Department of Rheumatology and Immunology, Yiwu Central Hospital, 519 Nanmen Street, Yiwu, Zhejiang Province 322000, P.R. China; 2 Yunyan Jin, Department of Rheumatology and Immunology, Yiwu Central Hospital, 519 Nanmen Street, Yiwu, Zhejiang Province 322000, P.R. China; 3 Dongni Zhao, Department of Rheumatology and Immunology, Yiwu Central Hospital, 519 Nanmen Street, Yiwu, Zhejiang Province 322000, P.R. China; 4 Lixia Yu, Department of Rheumatology and Immunology, Yiwu Central Hospital, 519 Nanmen Street, Yiwu, Zhejiang Province 322000, P.R. China; 5 Huankun Cao, Department of Ultrasound, Yiwu Central Hospital, Yiwu, Zhejiang Province 322000, P.R. China

**Keywords:** Disease modifying anti-rheumatic drugs, Glucocorticoid, Arthritis, Combined

## Abstract

**Objectives:**

To explore the effect of glucocorticoids combined with disease modifying anti-rheumatic drugs in the treatment of symptoms in patients with rheumatoid arthritis.

**Methods:**

Medical records of patients with rheumatoid arthritis treated in the Rheumatology and Immunology Department of Yiwu Central Hospital from March 2020 to March 2021 were selected. A total of 38 patients were treated with disease modifying anti-rheumatic drugs Group-I and 44 patients were treated with disease modifying anti-rheumatic drugs and glucocorticoids Group-II. The symptom improvement of the two groups were compared and analyzed Serological indexes and adverse reactions.

**Results:**

Swollen joint counts (SJC), tender joint counts (TJC), rheumatoid arthritis disease activity evaluation form (DAS28) score, erythrocyte sedimentation rate, levels of ESR, C-reaction protein (CRP) and rheumatoid factor (RF) of Group-II patients were lower than those in Group-I (P<0.05). The adverse reaction rate in Group-II patients was 12.20%, which was similar to that of Group-I patients. There was no significant difference in 9.76% of the patients (P>0.05).

**Conclusion:**

The combination of glucocorticoids and disease modifying anti-rheumatic drugs in the treatment of patients with rheumatoid arthritis is safe can further improve their symptoms and serological indexes, and will not lead to increased adverse reactions.

## INTRODUCTION

Rheumatoid arthritis is a common systemic autoimmune disease that mainly manifests as systemic arthritis. Its pathological characteristics are mainly vasculitis and synovitis. Rheumatoid arthritis can lead to bone destruction, and may affect ligaments, tendons, muscles and other tissues around the joint, resulting in decreased joint stability and joint deformity and dysfunction.^1^ Moreover, vasculitis can also affect the surrounding organs and tissues, including lungs, which can lead to life-threatening complications of pulmonary interstitial fibrosis.^2^ In recent years, the standardized systematic treatment of rheumatoid arthritis has been continuously emphasized in clinic. In addition to symptom relief and improvement of joint function, the treatment measures focus on delaying the progress of arthritis and preventing systemic damage. At present, rheumatoid arthritis is mainly treated by drugs. The commonly used drugs are anti-rheumatic drugs, biological agents, glucocorticoids, etc. These measures effectively control disease activity, prevent bone destruction process, and improve the overall long-term prognosis of patients.^3^ In the past, most patients with rheumatoid arthritis were treated with disease modifying anti-rheumatic drugs alone. However, this treatment alone is not able to effectively delay the progress of the disease. Glucocorticoids have significant anti-inflammatory effect and can regulate immune function and provide symptomatic relief.

However, dose increase is often necessary, leading to increased rate of adverse reactions.^4^ In recent years, our hospital has carried out the treatment of rheumatoid arthritis through the combination of glucocorticoids and disease modifying anti-rheumatic drugs. The main goal of this study was to further explore the safety and the effect of the combined treatment on symptoms and serological indexes of patients with rheumatoid arthritis.

## METHODS

The records of patients with rheumatoid arthritis treated in the Rheumatology and Immunology Department of Yiwu Central Hospital from March 2020 to March 2021 were collected. Of the 82 patients, 29 were male and 53 were female.

### Inclusion criteria:

- The diagnosis was made according to the diagnostic criteria of rheumatoid arthritis proposed by the American Society of Rheumatology (ACR);^5^
- In the active stage of disease;
- Complete medical history;
- Cooperate with informed consent.

### Exclusion criteria:

- There are serious immune diseases;
- Complicated with severe organ diseases;
- Severe systemic infection;
- Pregnant and lactating women.

This study was approved by the medical ethics committee of Yiwu central hospital (No. YWZX2021009, Date: 2021-03-18).

Disease modifying anti-rheumatic drug treatment scheme (Group-I) included methotrexate (Shanghai Shangyao Xinyi Pharmaceutical Co., Ltd., H31020644) 10mg/week, oral, once a day, five times/week, for three months; Leflunomide (Merro Pharmaceutical Co., Ltd., H20080047) 10mg/time, oral, twice a day for three months. Combination of glucocorticoids and disease modifying anti-rheumatic drugs therapy Group-II was as follows: on the basis of the regimen, described in Group-I, patients were administered prednisone acetate tablets (Tianjin Tian Yao pharmaceutical Limited by Share Ltd, H12020689). Initial dose was 10mg/day, oral. After stabilization of the patient’s condition, the adjusted dose was 3mg/day and was maintained for three months.

Basic information of patients was collected. After three months of treatment the following indicators were evaluated:

### Symptom improvement:

The number of swollen joints (SJC) and tender joint counts (TJC) in both groups were recorded respectively on the day of admission and the day of completion of the course of treatment. The disease activity of the two groups was evaluated through the rheumatoid arthritis disease activity evaluation form (DAS28). The score was 0~28. The higher the score, the higher the disease activity.^6^

### Serological indexes:

4ml fasting venous blood samples of the two groups were collected on the day of admission and the day of completion of the course of treatment. Levels of erythrocyte sedimentation rate (ESR), C-reaction protein (CRP) and rheumatoid factor (RF) were detected by immunoturbidimetry;

### Safety:

The incidence of adverse reactions such as abnormal liver function, leucopenia, gastrointestinal discomfort, hair loss and rash were counted.

### Statistical Analysis:

SPSS 22.0 was used to analyze and process the collected data, [n (%)] was used to represent the counting data, the test method was *x*^2^, (x̄±s) was used to represent the measurement data, t-test was used when the distribution was normal, and the test level was a = 0.05, while rank sum test was used when the distribution was not normal, (P<0.05) was considered statistically significant.

## RESULTS

A total of 82 patients (29 males and 53 females) met the inclusion criteria. The age ranged from 40 to 75 years, with an average of (58.08±10.26) years. The course of disease ranged from one to seven years, with an average of (4.22±1.6) years. 38 patients were treated with Group-I and 44 patients were treated with Group-II.

There was no significant difference in the basic clinical characteristics between the two groups (P>0.05) Table-I. On the day of admission, there was no significant difference in SJC, TJC and DAS28 scores between the two groups (P>0.05). On the day of treatment completion, SJC, TJC and DAS28 scores in both groups were lower than those on the day of admission. SJC, TJC and DAS28 scores were significantly lower in patients treated with Group-II as compared to Group-I (P<0.05) (Table-II). On the day of admission, there was no significant difference in ESR, CRP and RF between the two groups (P>0.05). On the day of completion of treatment, ESR, CRP and RF of the two groups decreased compared with the day of admission, and were significantly lower in patients treated with Group-II when compared to patients treated with Group-I (P<0.05) Table-III. The incidence of adverse reactions in patients treated with Group-II was 12.20%, similar (P>0.05) to that of patients, treated with Group-I (9.76%) Table-IV.

**Table-I:** Comparison of general information between the two groups.

Group	n	Gender (Male/Female)	Age (Year)	Course of disease (Years)
Group-I	38	16/22	57.97±10.32	4.47±1.57
Group-II	44	13/31	58.18±10.33	4.00±1.61
x^2^/t	-	1.407	0.091	1.341
P	-	0.236	0.928	0.184

**Table-II:** Comparison of symptom improvement between the two groups (x¯±s).

	SJC(Piece)				TJC(Piece)				DAS28 (score)			

Group (n)	Admission day	Treatment completion date	t	P	Admission day	Treatment completion date	t	P	Admission day	Treatment completion date	t	P
Group-I (n=38)	7.60±2.02	3.07±1.05	18.358	<0.001	9.52±2.22	3.81±1.06	20.071	<0.001	18.13±3.67	9.26±3.14	28.272	<0.001
Group-II (n=44)	7.56±2.07	1.79±0.85	22.948	<0.001	9.47±2.61	1.86±0.87	22.722	<0.001	18.14±3.43	6.20±2.18	38.814	<0.001
t	0.082	6.112	-	-	0.091	9.111	-	-	0.006	5.172	-	-
P	0.935	<0.001	-	-	0.928	<0.001	-	-	0.995	<0.001	-	-

**Table-III:** Comparison of serological indicators between the two groups (x¯±s).

Group (n)	ESR (mm/h)				CRP (mg/L)				RF (IU/ml)			

	Admission day	Treatment completion date	t	P	Admission day	Treatment completion date	t	P	Admission day	Treatment completion date	t	P
Group-I (n=38)	79.02±11.33	37.08±6.79	46.959	<0.001	37.60±5.62	19.10±5.79	65.113	<0.001	164.47±18.19	99.08±17.81	275.743	<0.001
Group-II (n=44)	77.95±11.13	28.22±4.19	41.107	<0.001	37.43±6.87	12.79±3.87	44.337	<0.001	163.02±17.33	62.93±16.43	291.128	<0.001
t	0.431	7.204	-	-	0.125	5.867	-	-	0.369	9.555	-	-
P	0.668	<0.001	-	-	0.901	<0.001	-	-	0.713	<0.001	-	-

**Table-IV:** Comparison of adverse reactions between the two groups [n (%)].

Group	n	Adverse reactions					Total

		Abnormal liver function	Leukopenia	Gastrointestinal discomfort	Hair loss	Rash	
Group-I	38	1 (2.44)	1 (2.44)	1 (2.44)	0 (0.00)	1 (2.44)	4 (9.76)
Group-II	44	1 (2.44)	1 (2.44)	1 (2.44)	1 (2.44)	1 (2.44)	5 (12.20)
x^2^	-	-	-	-	-	-	0.125
P	-	-	-	-	-	-	0.724

## DISCUSSION

Our study showed that the combination of disease modifying anti-rheumatic drugs and glucocorticoids was safe and more efficient in improving symptoms and serological indicators in patients with rheumatoid arthritis. Disease modifying anti-rheumatic drugs are commonly used in the treatment of rheumatoid arthritis, and can effectively alleviate the symptoms such as joint pain and swelling and reduce the disease activity. However, the effect of single application is not ideal.^7^ In recent years, studies have showed that the combination of disease modifying anti-rheumatic drugs and glucocorticoids can achieve good anti-inflammatory and immune regulation effects, and promote effective improvement of the prognosis in patients with rheumatoid arthritis.^8^

The results of this study showed that the SJC, TJC and DAS28 scores of patients treated with Group-II were lower than those treated with Group-I only (P<0.05), suggesting that the treatment of rheumatoid arthritis with disease modifying anti-rheumatic drugs combined with glucocorticoids can further alleviate the symptoms. Aletaha D et al.^9^ reported in the literature review that when methotrexate was used in combination with glucocorticoids, 40% to 50% of patients achieved remission or at least low disease activity. This is consistent with the results of this study. After the occurrence of rheumatoid arthritis, the large and small joints of patients can be subject to inflammatory infiltration, resulting in joint swelling, tenderness, movement disorder, etc., which seriously affects the daily life of patients.^10^

The disease modifying anti-rheumatic drug selected in this study was methotrexate, which belongs to a category of cell-cycle specific drugs. It can inhibit the proliferation and division of lymphocytes by reducing the activities of dihydrofolate reductase and formyl transferase, thus playing anti-inflammatory and anti-rheumatic role.^11^ Glucocorticoids can enter the cytoplasm, bind and activate glucocorticoid receptors, inhibiting inflammatory factors. In 2002, when the American Rheumatology Association revised the treatment guidelines for rheumatoid arthritis, it listed a glucocorticoid bonasone acetate as one of the therapeutic drugs. Numerous clinical studies showed that low-dose glucocorticoid treatment for patients with rheumatoid arthritis can quickly alleviate the related symptoms such as joint swelling and pain.^12^,^13^ Combined action of disease modifying anti-rheumatic drugs and glucocorticoids may, therefore, prove more effective to promote the remission of patients’ symptoms and reduce the activity of the disease.

ESR, CRP and RF are commonly used clinical efficacy evaluation indicators of rheumatoid arthritis, and their levels are positively correlated with disease severity, which can reflect disease activity and patient prognosis.^14^ Earlier, Kang X et al.^15^ observed that the levels of rheumatoid factor (RF) and C-reactive protein (CRP) decreased significantly in 20 patients after one month of treatment with disease modifying anti-rheumatic drugs and glucocorticoids. At the same time, Hua L et al.^16^ conducted a controlled experiment on the efficacy and safety of low-dose glucocorticoids (GCS) combined with total methotrexate (MTX) and hydroxyquine (HCQ) in rheumatoid arthritis patients one year after oral administration. The results showed that low-dose GCS combined with MTX and HCQ effectively promote disease remission, as indicated by ACR20 and DAS28-ESR scores, and improve clinical and radiological outcomes in patients significantly better than placebo+MTX+HCQ with no increase in adverse reactions. In agreement with these observations, in our study, the levels of ESR, CRP and RF in patients treated with Group-II were lower than those treated with Group-I (P<0.05), suggesting that the combination of disease modifying anti-rheumatic drugs and glucocorticoids in the treatment of patients with rheumatoid arthritis is helpful to further improve the serological indexes of patients.

Disease modifying anti-rheumatic drugs can effectively alleviate the joint damage of patients, but the effect is slow, and may take up to 3~6 months. Rapid and significant anti-inflammatory effect of glucocorticoids can, therefore, quickly improve symptoms, alleviate the disease, and promote the improvement of relevant serological indexes before the expected effect of disease modifying anti-rheumatic drugs takes place.^17^,^18^ In addition, our study also found that the incidence of adverse reactions in patients treated with Group-II was 12.20%, which was not statistically significant compared with 9.76% in patients treated with Group-I(P>0.05). These results are in agreement with the previous report by Mary safe Khan et al.^19^ that showed no statistically significant difference in the incidence of adverse reactions between GC users and non-GC users in a rheumatoid arthritis trial. It is suggested that glucocorticoid treatment on the basis of disease modifying anti-rheumatic drugs will not lead to the increase of treatment-related adverse reactions in patients with rheumatoid arthritis. However, glucocorticoid is known to affect ion, glucose and lipid metabolism, as well as cardiovascular and cerebrovascular system. In this study, the initial dose of prednisone acetate tablets was 10mg/day in the beginning and then adjusted to 3mg/day and maintained, with no obvious adverse reactions observed during the treatment.

### Limitations of the study:

It included small sample size (only 82 patients) with only few observation indicators. Data were recorded only until the end of the course of treatment, and there is high subjectivity, which may make the conclusions one-sided and limited. The follow-up study should select a large sample size, observe more relevant indicators and follow-up.

## CONCLUSION

In the treatment of rheumatoid arthritis, the combination of glucocorticoids and disease modifying anti-rheumatic drugs can effectively promote the remission of patients’ symptoms and improve relevant serological indexes, and is not associated with increased rate of adverse events.

### Authors’ contributions:

**LS:** Conceived and designed the study.

**YJ, DZ, LY & HC:** Collected the data and performed the analysis.

**LS:** Was involved in the writing of the manuscript and is responsible for the integrity of the study.

All authors have read and approved the final manuscript.

## References

[1] Pincus T, Castrejon I, Yazici Y, Gibson KA, Bergman MJ, Block JA (2019). Osteoarthritis is as severe as rheumatoid arthritis:Evidence over 40 years according to the same measure in each disease. Clin Exp Rheumatol.

[2] Wagan AA, Bhutoo AQ, Khan D, Raheem A (2020). Fatty liver in Pakistani cohort with rheumatoid arthritis. Pak J Med Sci.

[3] Gillespie J, Savic S, Wong C, Hempshall A, Inman M, Emery P (2012). Histone deacetylases are dysregulated in rheumatoid arthritis and a novel histone deacetylase 3-selective inhibitor reduces interleukin-6 production by peripheral blood mononuclear cells from rheumatoid arthritis patients. Arthritis Rheum.

[4] Brennan P, Harrison B, Barrett E, Chakravarty K, Scott D, Silman A (1996). A simple algorithm to predict the development of radiological erosions in patients with early rheumatoid arthritis:prospective cohort study. BMJ.

[5] Shresher NM, Mohamed AE, Elshahaly MH (2019). Performance of 2016 revised fibromyalgia diagnostic criteria in patients with rheumatoid arthritis. Rheumatol Int.

[6] Ng JY, Azizudin AM (2021). Quantity and Quality of Rheumatoid Arthritis and Osteoarthritis Clinical Practice Guidelines:Systematic Review and Assessment Using AGREE II. Curr Treat Options Rheum.

[7] Goldsmith CH, Boers M, Bombardier C, Tugwell P (1993). Criteria for clinically important changes in outcomes:development, scoring and evaluation of rheumatoid arthritis patient and trial profiles. OMERACT Committee. J Rheumatol.

[8] de Thurah A, Andersen IT, Tinggaard AB, Riis AH, Therkildsen J, Bøtker HK (2020). ?Risk of major adverse cardiovascular events among patients with rheumatoid arthritis after initial CT-based diagnosis and treatment. RMD Open.

[9] Aletaha D, Smolen JS (2018). Diagnosis and Management of Rheumatoid Arthritis:A Review. JAMA.

[10] Wagan AA, Raheem A, Bhatti A, Zafar T (2021). Fatigue assessment by FACIT-F scale in Pakistani cohort with Rheumatoid Arthritis (FAF-RA) study. Pak J Med Sci.

[11] Alpay-Kanitez N, Pehlivan O, Omma A, Can-Sandikci S, Girgin S, Icacan OC (2020). Favorable retention rates and safety of conventional anti-rheumatic drugs in older patients with rheumatoid arthritis. Medicine (Baltimore).

[12] Hua C, Buttgereit F, Combe B (2020). Glucocorticoids in rheumatoid arthritis:current status and future studies. RMD Open.

[13] Thompson WL, Abeles FB, Beall FA, Dinterman RE, Wannemacher RW (1976). Influence of the adrenal glucocorticoids on the stimulation of synthesis of hepatic ribonucleic acid and plasma acute-phase globulins by leucocytic endogenous mediator. Biochem J.

[14] Aghdashi MA, Seyedmardani S, Ghasemi S, Khodamoradi Z (2019). Evaluation of Serum Calprotectin Level and Disease Activity in Patients with Rheumatoid Arthritis. Curr Rheumatol Rev.

[15] Kang X, Wu Q, Wang K (2010). Efficacy of integrative medicine for the treatment of rheumatoid arthritis and its effect on glucocorticoid receptor expression. Zhongguo Zhong Xi Yi Jie He Za Zhi.

[16] Hua L, Du H, Ying M, Wu H, Fan J, Shi X (2020). Efficacy and safety of low-dose glucocorticoids combined with methotrexate and hydroxychloroquine in the treatment of early rheumatoid arthritis:A single-center, randomized, double-blind clinical trial. Medicine (Baltimore).

[17] Fiehn C (2019). S2e-Leitlinie „Therapie der rheumatoiden Arthritis mit krankheitsmodifizierenden Medikamenten “- was ist neu?. Arthritis Rheuma.

[18] Buttgereit F (2020). Views on glucocorticoid therapy in rheumatology:the age of convergence. Nat Rev Rheumatol.

[19] Safy Khan M, Jacobs JWG, de Hair MJH, Welsing PMJ, Edwardes MD, Teitsma XM (2020). Effect on efficacy and safety trial outcomes of also enrolling patients on ongoing glucocorticoid therapy in rheumatoid arthritis clinical trials of tocilizumab or adalimumab or methotrexate monotherapy. Ann Rheum Dis.

